# Enhancing the Longitudinal Compressive Strength of Freeform 3D-Printed Continuous Carbon Fiber-Reinforced Polymer Composite Laminate Using Magnetic Compaction Force and Nanofiber Z-Threads

**DOI:** 10.3390/ma17071589

**Published:** 2024-03-30

**Authors:** Mohammad Rakibul Islam, Md Nazim Uddin, Wyatt Taylor, Ryan Warren, Kuang-Ting Hsiao

**Affiliations:** 1 Department of Mechanical Engineering, University of South Alabama, Mobile, AL 36688, USA; mi2121@jagmail.southalabama.edu (M.R.I.); mu1621@jagmail.southalabama.edu (M.N.U.); wwt1621@jagmail.southalabama.edu (W.T.); raw1522@jagmail.southalabama.edu (R.W.); 2 Program of Systems Engineering, University of South Alabama, Mobile, AL 36688, USA

**Keywords:** magnetic compaction force-assisted additive manufacturing (MCFA-AM), 3D printing, carbon fibers, nanoparticles, polymer–matrix composites (PMCs), void

## Abstract

Low fiber-direction compressive strength is a well-recognized weakness of carbon fiber-reinforced polymer (CFRP) composites. When a CFRP is produced using 3D printing, the compressive strength is further degraded. To solve this issue, in this paper, a novel magnetic compaction force-assisted additive manufacturing (MCFA-AM) method is used to print CFRP laminates reinforced with carbon nanofiber (CNF) z-threads (i.e., ZT-CFRP). MCFA-AM utilizes a magnetic force to simultaneously levitate, deposit, and compact fast-curing CFRP prepregs in free space and quickly solidifies the CFRP laminate part without any mold nor supporting substrate plate; it effectively reduces the voids. The longitudinal compressive test was performed on five different sample types. ZT-CFRP/MCFA-AM samples were printed under two different magnetic compaction rolling pressures, i.e., 0.5 bar and 0.78 bar. Compared with the longitudinal compressive strength of a typical CFRP manufactured by the traditional out-of-autoclave–vacuum-bag-only (OOA-VBO) molding process at the steady-state pressure of 0.82 bar, the ZT-CFRP/MCFA-AM samples showed either comparable results (by −1.00% difference) or enhanced results (+7.42% improvement) by using 0.5 bar or 0.78 bar magnetic rolling pressures, respectively.

## 1. Introduction

### Motivation

Fiber-reinforced composites have become increasingly attractive materials for use in the aerospace, automotive, and sports industries primarily from their exceptional strength and their low weight. These composites also present a remarkable opportunity for customizable properties according to the requirements of specific applications. Various factors play a role in altering composite properties. These include the fiber type and matrix composition, along with their respective volume fractions and stacking schemes. Moreover, the interface between the fiber and the resin matrix holds great significance for influencing the properties of composite materials. Methods for interface modification include fiber surface roughening, interphase formation, and surface coating. The growth of nano species, in this case, nanotubes/nanofibers on the fiber surface, has also been used to modify the surface interface [1]. CNFs are a particularly promising reinforcement for composites due to their light weight, enhanced durability, high Young’s modulus, and high aspect ratio.

Numerous studies investigated how various types of nanofillers as reinforcements on composites could attain better mechanical and electrical properties. CNFs are known to possess excellent mechanical strength with Young’s modulus and the tensile strength of approximately 600 GPa and approximately 8.7 GPa, respectively [2]. The Young’s modulus for CNF is approximately three times that of steel. In their study, Liu et al. [3] investigated the impact of different filler materials and their ratios on the flexural and compressive properties of composites by reinforcing the matrix with halloysite and nano silica. A control sample was made using the vacuum-assisted resin infusion molding (VARIM) method to compare. All laminates were produced using unidirectional T300 carbon fiber, and the matrix contained nanofillers at varying concentrations, ranging from 1% to 20% by weight, and consisted of piperidine as a hardener and epoxy resin (Araldite-F from the Huntsman company). After testing, the liquid rubber nanofiller was found to have minimal effect on the flexural and compressive properties. Zhou et al. [4] employed VARTIM to produce a satin weave carbon/epoxy composite incorporated with CNFs. The epoxy resin matrix was changed by adding 2 wt% of CNFs to enhance the matrix-dominated properties of the composite. As a result of this modification, the compressive strength of the composite had nearly a 20% improvement from 292 MPa to 350 MPa while maintaining a fiber volume fraction (FVF) of 56%. Microscopic analysis of the composite revealed instances of crack bridging and reduced crack openings, which were attributed to the addition of 2 wt% CNFs in the matrix resin. These findings indicate that the inclusion of CNFs improved the composite’s compressive strength and crack resistance. Iwahori et al. [5] used the hot-press method to produce a composite part, which included a modified resin blend (both 5 wt% and 10 wt% CNFs in EPIKOTE 827 resin, respectively) to impregnate plain weave TORAYCA C6343 fabric and succeeded in enhancing the interlaminar strength and improving the compressive strength of the CNFs-infused CFRP laminate by about 15%.

Anand et al. [6] conducted a study on the resin film infusion (RFI) method to incorporate randomly aligned multiwalled carbon nanotubes (MWCNTs) into an epoxy matrix, aiming to enhance various properties of unidirectional E-glass composites. The compressive strength of the MWCNT-modified samples exhibited a substantial increase from 620 MPa to 770 MPa, representing an enhancement of approximately 24% compared to the control E-glass samples. In another research by Sharma and Lakkad [7], a thermal chemical vapor deposition (CVP) process was utilized to grow MWCNTs on the carbon fabric’s surface at 700 °C. The compressive strength of it demonstrated a remarkable improvement, with approximately a 67% and 4% increase in the transverse direction and longitudinal direction, respectively, over the control sample. The control case was prepared using similar thermal treatment and compression die molding techniques without MWCNTs.

Molding, injection, and extrusion are examples of traditional composite manufacturing processes. Molding processes are considered the most suitable for the manufacturing of complex-shaped C-CFRP (continuous-carbon fiber reinforced polymer) parts. Traditional molding methods include out-of-autoclave–vacuum-bag-only (OOA-VBO) molding, reaction injection molding, resin film infusion, resin transfer molding, compression molding, and elastic reservoir molding. The molded part’s mechanical properties depend on factors such as raw material quality, fiber alignment precision, degree of cure, and molding defects. Molded part quality improvement involves careful consideration of part and mold designs to prevent the formation of dry spots and voids, although this may not eliminate voids completely [8]. Furthermore, regular inspection of molded parts is crucial to avoid a premature failure. Molding processes require significant preparation efforts and extended manufacturing times. These processes are followed by periods of post cleaning, mold tooling storage, and overall maintenance. Thus, these additional steps can contribute to an increase in the overall production complexity and time consumption.

Despite the processing conveniences and advantages that current 3D printing technologies offer in comparison to traditional manufacturing methods, most 3D printing methods still fall short of producing C-CFRP parts with the same level of strength compared to those through traditional methods. Table 1 provides a comparison of the published data [9,10,11] on C-CFRP parts manufactured through improved 3D printing methods with those produced using traditional composite manufacturing methods and typical FDM 3D printing.

As seen in Table 1, there is an absence of detailed longitudinal compressive strength data of 3D-printed CFRP composites in the reported literature the authors can find. However, the lower ILSS (interlaminar shear strength) of 3D-printed C-CFRP could also indirectly indicate a likely lower longitudinal compressive strength since they are all considered matrix-sensitive properties and susceptible to voids in the C-CFRP laminates. A new set of data from a recent paper [11] has been added to the table where the ILSS change vs. baseline value is respectively +0.37% and +11.34% for CFRP and ZT-CFRP samples printed by the novel MCFA-AM method.

From the literature analysis, one can hypothesize two possible routes to improve the longitudinal compressive strength of 3D-printed C-CFRP laminates: (i) improve the C-CFRP’s compressive strength with nanoparticles such as CNF and (ii) improve the void content control of the 3D printing process when handling C-CFRP. Further background information on this approach could help to judge the feasibility of this integrated approach to obtain high longitudinal compressive strength from 3D-printed C-CFRP.

Kirmse et al. [12] used the traditional OOA-VBO method to produce CFRP and CNF-modified CFRP laminates and reported that the 1 wt% CNF Z-threaded CFRP (i.e., ZT-CFRP) laminates had an improvement of approximately 25% (from 619.84 MPa to 773.76 MPa) and 15% (673.85 MPa to 773.76 MPa) in terms of longitudinal compressive strength when compared to unaligned 1 wt% CNF-modified CFRP (UA-CFRP) laminates and control CFRP laminates, respectively. The improvement in the longitudinal compressive strength of composites was achieved through the threading of low-dosage (only 1 wt% into the resin matrix) CNFs in a zigzag pattern through the carbon fiber arrays along the z-direction (i.e., out-plane direction of the laminate). This threading mechanism creates a network of interlocked reinforced fibers, providing additional support against interlaminar and intralaminar carbon fiber buckling under longitudinal compressive loads, which is a primary cause of composite failure under compression.

In a recent paper, the novel magnetic compaction force-assisted additive manufacturing (MCFA-AM) method combined with ZT-CFRP technology was utilized to print samples that yielded an improvement of 11.34% in ILSS when compared to benchmark CFRP samples produced with the traditional OOA-VBO molding method [11]. It is hypothesized for this paper that (i) CNF z-threads exhibit an influence on decreasing the void content of a sample, (ii) the compressive strength of CFRPs is enhanced due to the reinforcement of CNF z-threads, (iii) the CNF z-threads interlock the carbon fibers and secure them together, hence meaning the z-threading enhances the resistance to crack propagation by delaying and raising the threshold for microcrack growth, and (iv) a similar approach to aforementioned paper [11] will be adopted in this study to discover any improvement potential for the compressive strength of CFRP composites with increased compaction pressure using this technology.

**Figure 1:**
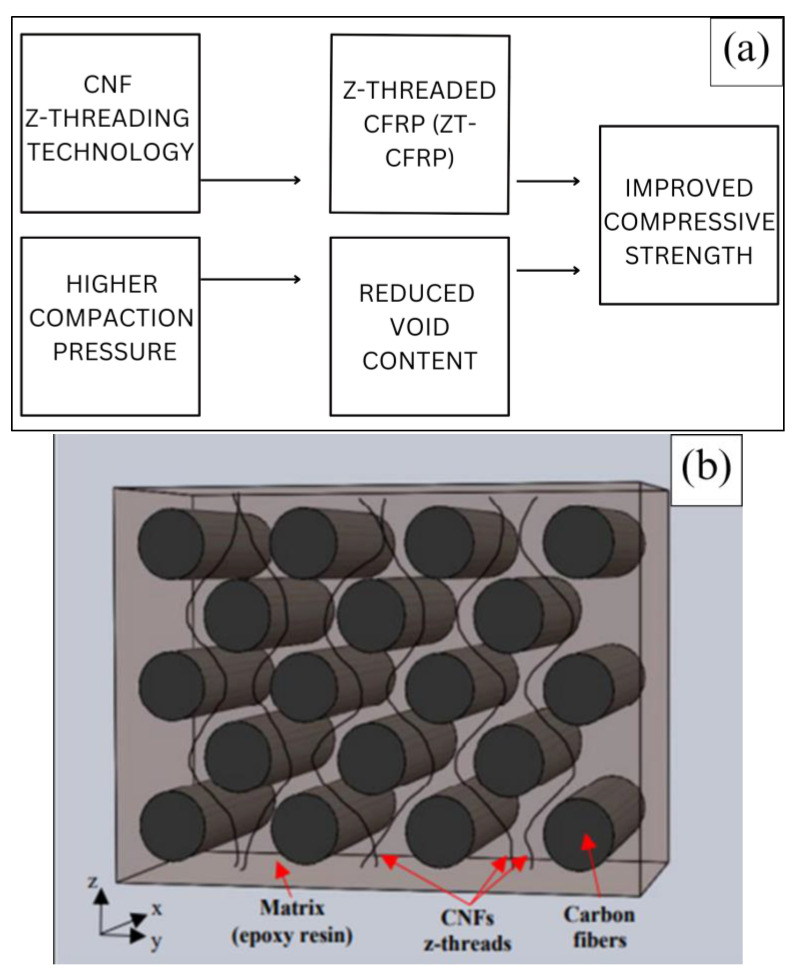
(**a**) Parameters for achieving improved compressive strength. (**b**) Illustration of ZT−CFRP structure (obtained from [11] with permission under open access Creative Commons CC BY 4.0 license).

In traditional manufacturing methods, specific processing parameters are commonly employed to achieve optimal results. For instance, high compression pressure is often used to effectively compress the prepreg laminas together, while a high vacuum is applied to remove any gas or volatile substances, which could lead to void formation in the final laminate. Additionally, these methods usually follow uniform and gradual control cycles during the resin curing process. This careful control of temperature allows one to minimize process-induced residual stress in the CFRP parts. Manufacturers aim to produce CFRP components with improved structural integrity and reduced defects by adhering to these established techniques. Figure 1 illustrates the parameters hypothesized to enhance the compressive strength of the composite through the innovative MCFA-AM method in this study. It provides a basic representation of the ZT-CFRP structure, showcasing CNFs threaded in the z-direction through carbon fiber arrays, forming a mechanically interlocked 3D fiber-reinforcement network. The use of a higher compaction pressure to reduce void content and the integration of advanced nanocomposites like ZT-CFRP, when combined appropriately, are expected to generate synergistic effects, significantly improving the overall quality and strength of the composite laminate.

**Figure 2:**
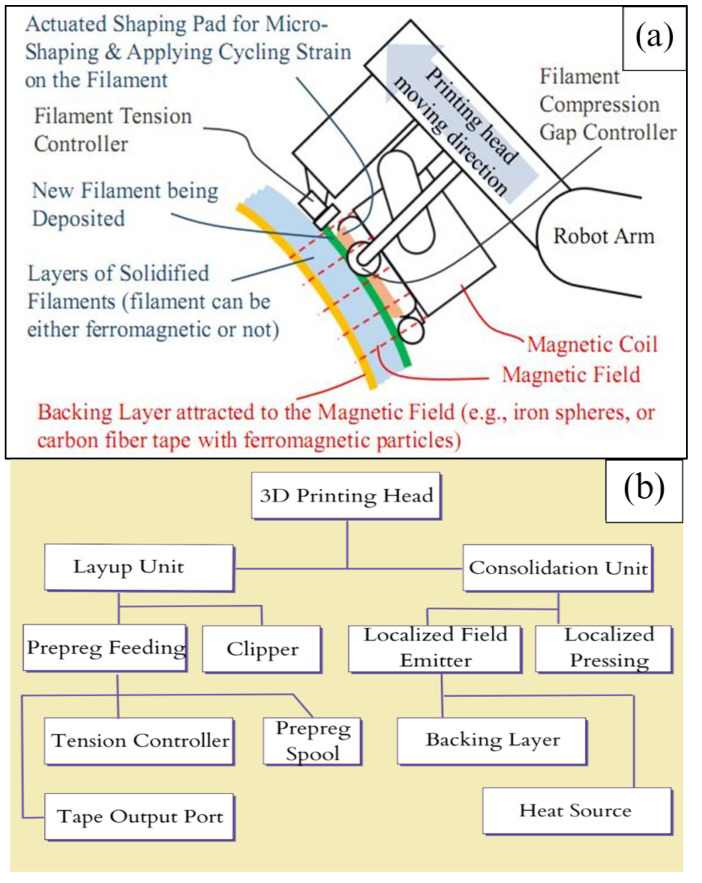
(**a**) Depiction of the novel MCFA−AM printhead (redrawn based on patent [13]). (**b**) A system construction map explaining the main printhead components and their relations.

The patented MCFA-AM 3D printing technique [13] utilizes the attractive force generated between a magnetic field emitter and a magnetic backing article for the rapid printing, compaction, and support of C-CFRP parts in open space, in turn eliminating the need for any molds. Through directing the movements of the MCFA-AM printhead with a robotic arm and using fast-curing C-CFRP prepregs, the swift production of C-CFRP parts in various sizes with precise intricacy, functionality, and improved strength can be achieved. Unlike traditional molding processes and conventional 3D printing methods, this approach requires neither a mold assembly nor a supporting printer bed. This results in minimal manufacturing size restrictions along with potentially reduced preparation and cleaning times. Moreover, the controllable magnetic compaction force greatly reduces the likelihood of void, air trap, and defect formation during C-CFRP part printing. The schematic of the novel MCFA-AM printhead proposed in its patent publication [13] and an accompanying system construction map showing the constituent components and their relationships are provided in Figure 2.

Void content can be a big matter of concern while using these traditional methods for producing composite laminates. The presence of voids can primarily stem from any kind of molding defects, a lack of adequate vacuum pressure in the case of the OOA-VBO method, the formation of internal residual stress, etc. They act as stress concentrators and weak points in the composite material, causing heterogeneous stress distributions, lower fatigue tolerance, decreased stiffness, and increased material instability. The presence of voids and agglomerates notably deteriorates the mechanical and electrical properties of composite parts. [14,15]. Qinghao He et al. [16] conducted a study where CM (compression molding) was adopted to highlight the impact of voids on the mechanical performance of 3D-printed CF/PA6 (Polyamide 6) composite laminates. The CM method helped reduce the void content to 6% from 12%, and the transverse tensile strength and flexural strength experienced remarkable improvements, increasing by 78% and 93%, respectively. When a compressive load is applied, these voids can lead to the formation of local stress concentrations, cracking, or delamination, causing premature failure and a reduction in compressive strength. In the MCFA-AM method, the localized pressing (in the consolidation unit) ensures the elimination of any air gap or void being formed in the interlaminar region and thus reduces the overall void content of the laminate.

In this study, a comparative experimental longitudinal compressive strength analysis was conducted between the MCFA-AM printed ZT-CFRP samples and C-CFRP samples produced through various methods, including MCFA-AM, OOA-VBO, and traditional FDM (where prepreg tape is fused without compaction pressure). Moreover, ZT-CFRP samples were produced using the conventional OOA-VBO molding method. The longitudinal compressive strength of all sample types was assessed using a TINIUS OLSEN testing machine. Subsequently, microscopic images were captured to examine fracture sites and voids in the samples.

## 2. Materials and Methods

### 2.1. Materials

Unidirectional (UD) HexTow^TM^ AS4 carbon fiber fabric with a fiber density of 1.79 g/cm^3^, an areal weight of 190 g/m^2^, and a 3k tow size, was employed as the feedstock filament for the MCFA-AM 3D printing experiment to prepare ZT-CFRP prepreg tapes. The PR-24-LD-HHT CNFs used in this study were sourced from Pyrograf Products (Applied Sciences, Inc., Cedarville, OH, USA). The resin blend consisted of EPON 862 (Miller Stephenson Chemical Co., Inc. Danbury, CT, USA), and Araldite LY 3031 (Huntsman Corp., Basel, Switzerland) at a ratio of 2:1, respectively. To ensure proper dispersion of the CNFs into the resin blend [8,11], Disperbyk-191 and Disperbyk-192 surfactants (BYK, Wallingford, CT, USA) were added. The matrix curing agent, Aradur 3032 from Huntsman Corp., was used at a ratio of 100:11 (resin blend/curing agent). This fast-curing agent initiates its cure cycle at 140 °C.

### 2.2. Testing Samples

Five different types of laminate samples were manufactured and tested in this study: Type 1 (ZT-CFRP/MCFA-AM), Type 2 (CFRP/MCFA-AM), Type 3 (ZT-CFRP/OOA-VBO), Type 4 (CFRP/OOA-VBO), and Type 5 (CFRP/no-pressure 3DP). Type 5 samples were produced via a method equivalent to FDM printing where no compaction pressure is applied while depositing feedstock layers. The procedures followed for prepreg production, the OOA-VBO process, and the MCFA-AM 3D printing process are detailed in the subsequent sections. Five specimens were tested for each type of sample.

### 2.3. Longitudinal Compression Strength Testing Method

**Figure 3:**
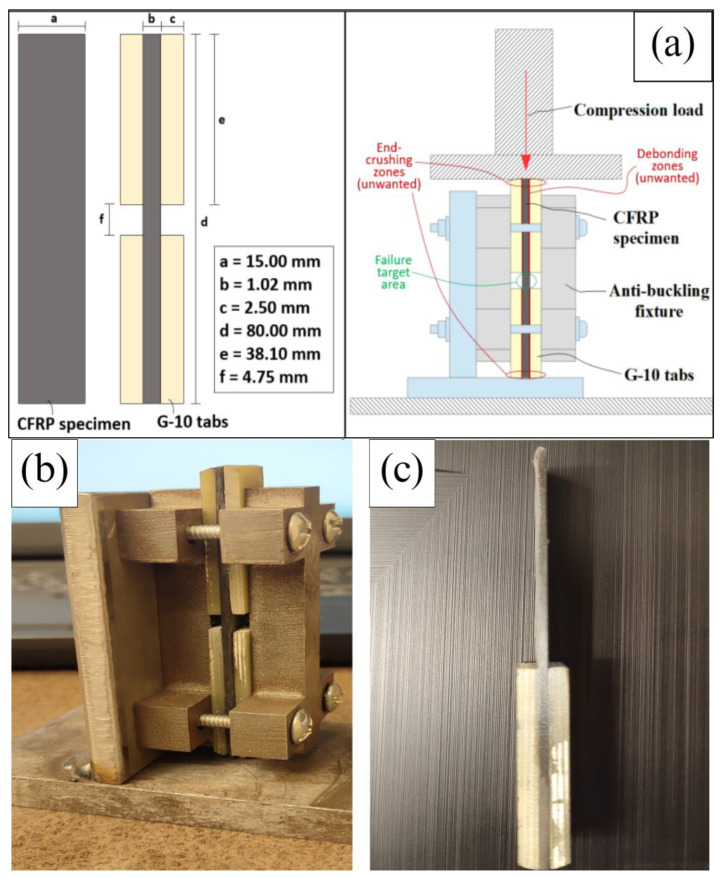
(**a**) Specimen dimension and anti-buckling testing setup. (**b**) Actual anti−buckling fixture. (**c**) Actual specimen.

The longitudinal compression strength test was conducted following a modified version of ASTM D695 [17], specifically using SRM 1R-94 [18], which pertains to the compression properties of oriented fiber–resin composites. SRM standards are developed by SACMA (Suppliers of Advanced Composite Materials Association). An anti-buckling fixture was constructed in accordance with the instructions provided in the SACMA standard to position the samples securely during testing. The fixture ensured that the samples remained stable and in place during the compression test. For the testing process, a TINIUS OLSEN Super “L” universal testing machine was utilized, equipped with a 12,000 lbf (53,379 N) load cell. The crosshead loading rate was set at 1.0 mm/min. To ensure a consistent and accurate loading alignment, and to avoid any eccentric strain or buckling, the top edges of the sample specimens were sanded flat. This step was taken to achieve a uniform distribution of the compression load during testing. To assess the mechanical properties, five specimens were tested for each type of laminate under consideration. Figure 3 provides depictions and photographs of the testing setup for reference.

### 2.4. Fast-Curing ZT-CFRP Prepreg and Laminates Printed Using MCFA-AM Method

To thread the CNFs into the fiber fabric in the z-direction, the patented radial flow alignment (RFA) technique [19] was used. The RFA technique utilizes converging radial flow rheology which causes the CNFs to stretch, align, and thread through the fiber fabric [19]. The dispersion of CNFs within the resin matrix is often challenging due to their tendency to agglomerate as a result of the Van der Waal forces acting on them. To counteract this, the CNFs were added into the Epon 862 resin along with BYK-191 and BYK-192 using high-shear mixing and sonication to ensure uniform dispersion following the process described in [11]. The Araldite LY 3031 epoxy and Aradur 3032 curing agent were then mixed into the solution to complete the fast-curing epoxy resin blend with CNF reinforcement. Within a fume hood, the mixture underwent high-shear mixing (HSM) at approximately 300 RPM for an hour, alternating directions every 30 min. The sonication of the blend was performed for one hour at a temperature of 90 °C. A quality control sample was observed under microscopic analysis to ensure there were no CNF agglomerates in the resin blend after these processes.

**Figure 4:**
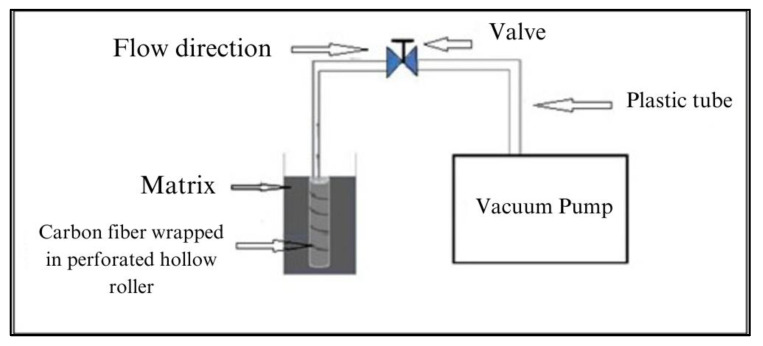
Radial flow alignment (RFA) process to manufacture the ZT−CFRP prepreg (obtained from [11] with permission under open access Creative Commons CC BY 4.0 license).

**Figure 5:**
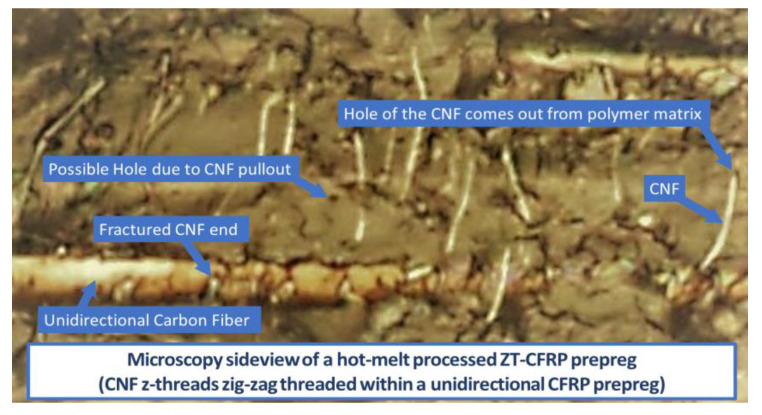
A microscopic sideview of a cured unidirectional ZT−CFRP prepreg shows the CNFs z−threading among the AS4 carbon fibers.

The curing agent was then promptly mixed into the fast-curing resin blend to maintain an optimal matrix viscosity and extend the cure life. The beaker containing the resin was then placed inside a 10 °C ice-water bath to allow adequate time to carry out the process. Figure 4 provides a schematic representation of the RFA process described in [11]. In this process, a carbon fiber fabric tape was wrapped around a hollow perforated tube connected to a vacuum pump. This unique setup created the converging radial flow of the CNF/resin blend and effectively threaded the CNFs into the carbon fiber fabric tape. Figure 5 shows the microscopic sideview of a cured unidirectional ZT-CFRP prepreg with CNFs z-threading within the array of AS4 carbon fibers after performing RFA.

**Figure 6:**
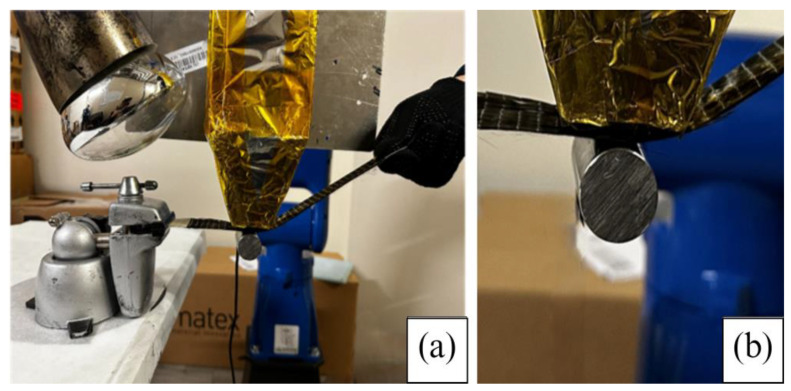
(**a**) The current iteration of the robotic arm-driven MCFA−AM printer. A tape-like feedstock (e.g., ZT−CFRP prepreg) is being hand-fed (an automated mechanical feeder is currently being developed) and held in place by a backing article (metallic rod) attracted towards a proprietary magnetic field emitter (sheathed by aluminum foil within the image) with a heat lamp (not turned on in these images) focused towards the compaction region. (**b**) Image showing the how the feedstock material is compressed and held by the compressive force generated between the backing article and magnetic field emitter (pictures obtained from [11] with permission under open access Creative Commons CC BY 4.0 license).

The ZT-CFRP and CFRP laminates were printed using a custom-made robotic MCFA-AM 3D printer. A printhead capable of performing MCFA-AM sat atop a 6-DOF robotic arm. The robotic arm was a Motoman GP 7 Robot Arm by Yaskawa America, Inc. (Waukegan, IL, USA). The robotic arm controlled the printhead’s position along a pre-determined linear path for printing. Magnetic compaction pressure was exerted onto the prepreg to print the samples using the following steps:

1. A ply of prepreg was fixed at one end. The printhead was employed with the magnetic field emitter activated at the desired strength to create an attractive force between itself and the backing article. The robot arm then smoothly drove the printhead along a predetermined path, simultaneously positioning, compacting, and curing (solidifying) the prepreg tape. All of these operations took place without a mold (see Figure 6).
2. To release the backing article, the magnetic field was reduced (for the current still-developing prototype, the backing article was manually collected, but future fully developed MCFA-AM printers will have a built-in collection unit). A new layer of prepreg was loaded onto the layer preceding it. The magnetic field was then recalibrated to the desired applied force to attract the backing article and compress the current layer against the previous layer.
3. Once the magnetic field emitter was activated, the printing head was driven along the path by the robot arm. During this process, the printing head deposited, compacted, and cured a new layer of prepreg tape on top of the previous layer.
4. Steps (2) and (3) were repeated until the prepreg stack reached the desired thickness of the completed laminate part.

The curing temperature applied to the prepreg tape through the use of a 250-Watt heat lamp was 140 °C. The lamp was positioned in such a way to focus on the location at which the compaction force was applied onto the prepreg tape. Prior to conducting the longitudinal compressive tests, the laminates underwent a post-curing process lasting 4 h to ensure complete resin curing. It is worth noting that this robotic MCFA-AM printer design is also compatible with thermoplastic CFRP tapes.

Figure 6 shows the current prototype of the automated MCFA-AM printer. The feedstock (e.g., CFRP or ZT-CFRP prepreg tape) was anchored on one end, while the other end was hand-fed. The prepreg tape was held and compressed between the magnetic field emitter arrangement and backing article. A closer image of how the feedstock material is held and compressed between the emitter and backing article is also provided.

### 2.5. OOA-VBO Molding Process

**Figure 7:**
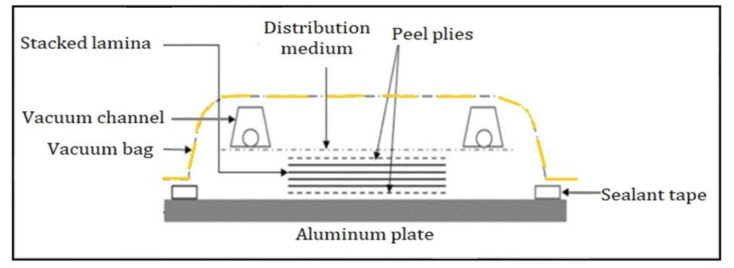
OOA−VBO layout scheme used for the preparation of laminates (obtained from [11] with permission under open access Creative Commons CC BY 4.0 license).

The OOA-VBO method was used to manufacture both CFRP and ZT-CFRP samples for testing. The OOA-VBO process took approximately 2 h to set the mold up, cure in between hot plates (to replace an oven), and clean afterwards. Figure 7 illustrates the setup followed for the OOA-VBO method, and the method is further described in [11,12]. Onto the aluminum plate (i.e., mold), a layer of peel ply was placed beneath the prepreg stack. Another layer of peel ply was added to the top of the stack before adding a layer of distribution medium (DM). Vacuum tubes were placed on top of the DM and used to seal the entire assembly within a vacuum bag. The vacuum bag held a pressure of 0.82 bar. The curing cycle used is presented in Table 2.

### 2.6. No-Pressure CFRP Laminate

To make the CFRP laminate cured with no compaction pressure to simulate FDM printing, there was neither a magnetic compaction force nor a vacuum applied onto the prepreg stacks. Instead, the prepreg was gently pressed by a hand roller to deposit each layer straight on the prepreg stack. A heat gun was used on the deposited stack at a temperature of 140 °C to initiate curing. Prior to ILSS testing, all sample types underwent an additional 4 h of curing to ensure that the specimen had reached full cure and would yield fair experimental comparisons.

## 3. Results and Analysis

The quantity of fiber present in a fiber-reinforced composite (FRP) has a direct correlation with some of the composite’s properties. In theory, a higher fiber volume fraction (FVF) leads to nearly linearly increased tensile strength, although this may not hold true for the compressive strength of the composite. The compressive strength, highly susceptible to fiber buckling failure micromechanics, is also significantly influenced by factors such as the matrix material, presence of voids, and the bonding between the matrix and fibers, and even a few degrees of the off-axial angle of the carbon fiber misalignment [20], especially if no nanoparticle reinforcement is considered. Equation (1) can be applied to calculate the fiber volume fraction (FVF) of an FRP prepreg:

(1) $$FVF=\frac{No.\;of\;plies\;of\;fabrics\times A_{\omega }}{\rho_{f}\times t_{lam}}$$

where Aω = areal weight of a fabric ply in g/cm^2^, ρ_f_ = density of fiber in g/cm^3^, and t_lam_= thickness of laminate in cm. The thickness of each prepreg layer can be determined as t_lam_/number ofpliesoffabrics. AS4 carbon fiber was employed in this study, with the fabric’s areal weight and fiber density as 0.019 g/cm^2^ and 1.79 g/cm^3^, respectively (per the manufacturer’s specifications). Ensuring a consistent fiber volume fraction (FVF) of 53% for each prepreg used in the MCFA-AM method was achieved by employing a gage-controlled conduit. This method involved carefully sliding fully impregnated prepregs through the conduit, serving two primary purposes: firstly, it effectively removed any excess resin, and secondly, it regulated and controlled the final thickness of the prepreg. To determine the void content, a random sample was selected from each laminate type. Three distinct microscopic images were captured for each sample type at a single location along the sideline. These locations were marked using a black marker pen, whose ink reflected differently from the resin and carbon fiber under the microscope. This approach aimed to enhance the visibility of voids by distinctly highlighting their presence (areas not painted with the marker pen) in the samples. Each void was assumed to be elliptical, characterized by a major axis length ‘a’ and a minor axis length ‘b’. The area of each void was divided by the total area of an individual microscopic spot to calculate the final void content in percentage.

**Figure 8:**
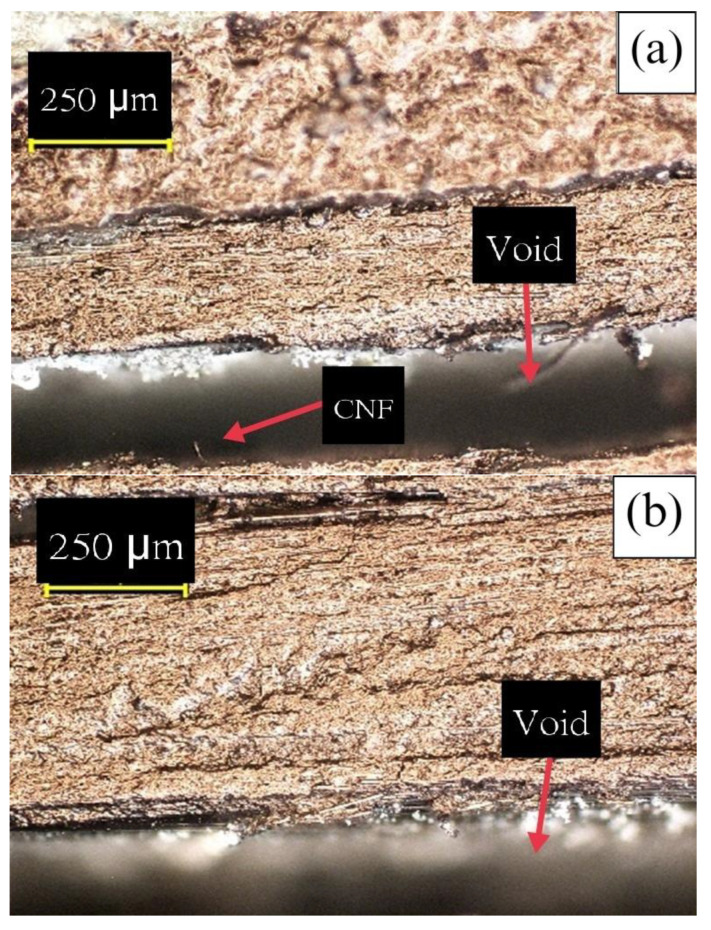

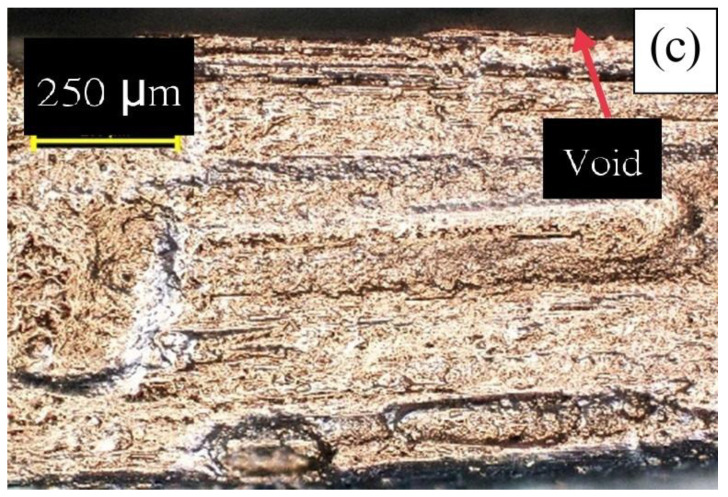
Microscope images (100×) of no−pressure 3DP CFRP sample with voids: (**a**) top line, (**b**) midline, (**c**) bottom line.

**Figure 9:**
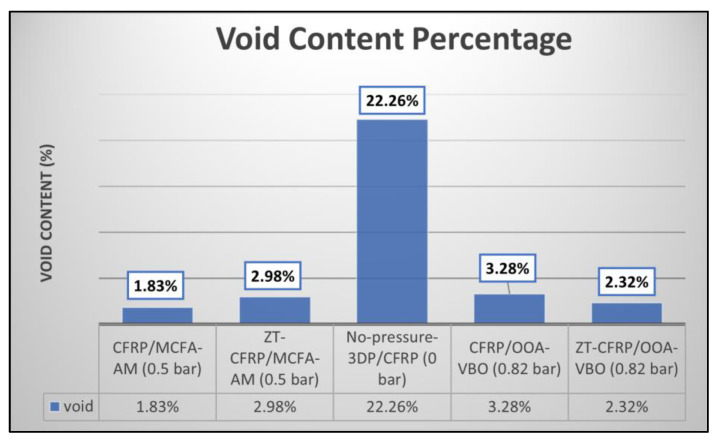
Void content plot for all different laminate types.

This method facilitated the accurate quantification of void content and the assessment of void presence and distribution in the laminate samples. Figure 8 displays images taken under an optical microscope (Nikon Eclipse LV150 Digital Sight DS-Fi1) at 100 times combined optical magnification, showcasing void spots in a no-pressure 3DP sample. The no-pressure 3DP CFRP samples exhibited the highest number of voids since they were printed without compaction pressure, resulting in a void content of approximately 22.26% with respect to the entire image area. Similar images were captured for other sample types to generate a comparison plot (Figure 9), illustrating void contents as percentages for all laminate types.

### 3.1. Compressive Strength Testing

**Figure 10:**
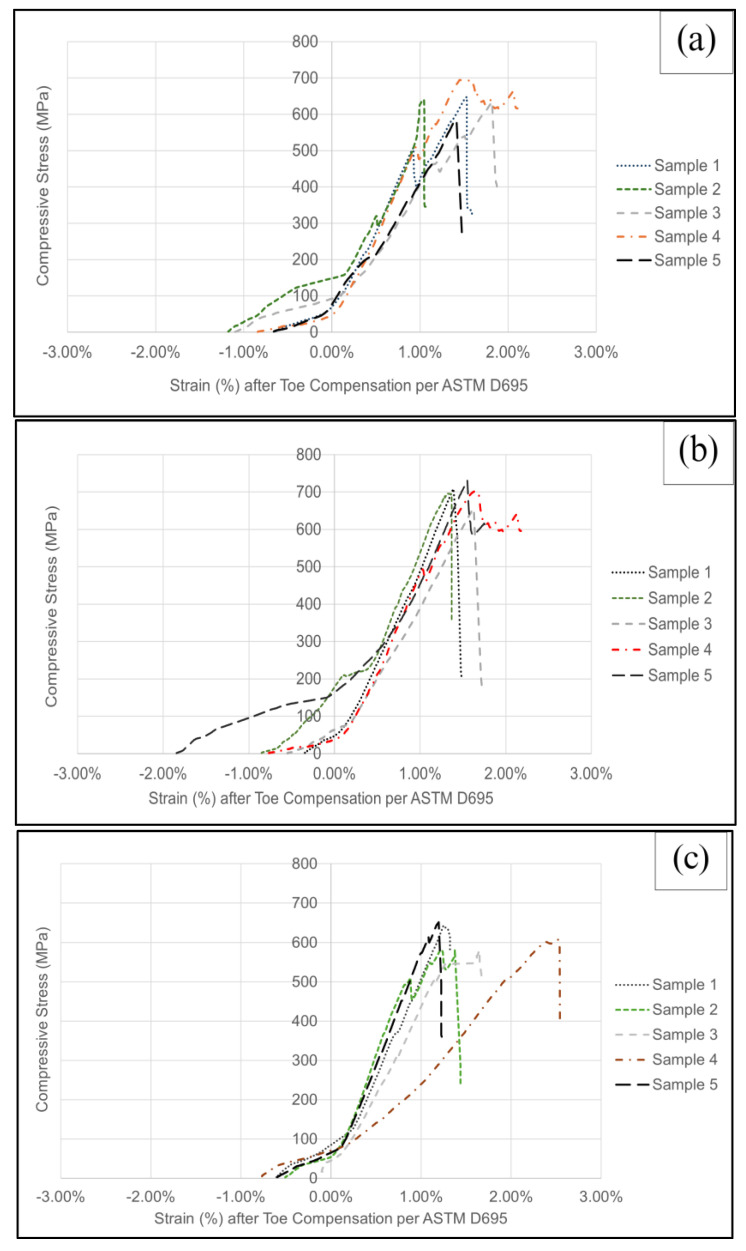

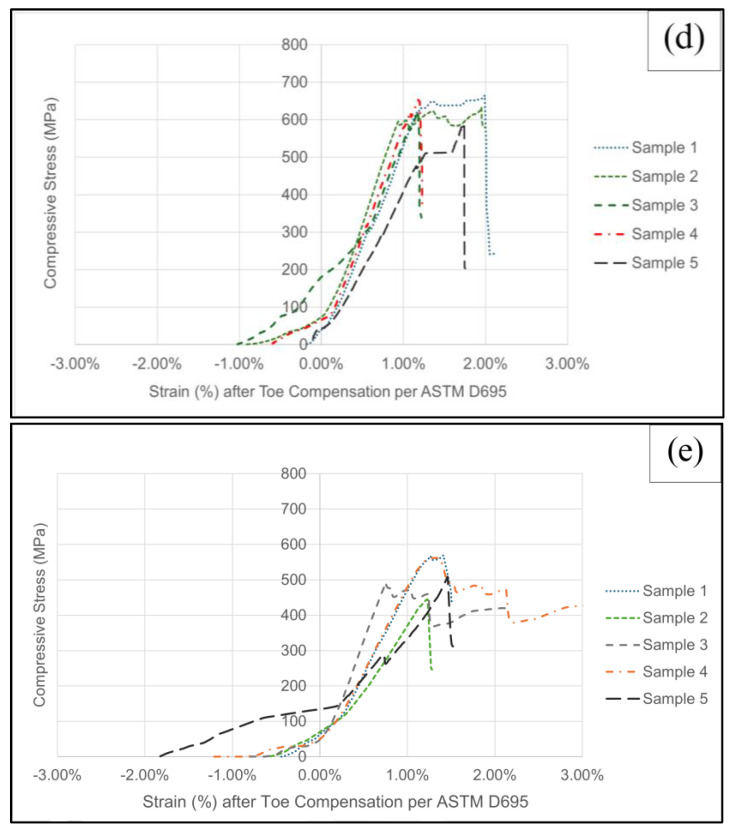
Longitudinal compressive stress (MPa) vs. strain plots. (**a**) Control CFRP/OOA−VBO. (**b**) ZT−CFRP/OOA−VBO. (**c**) CFRP/MCFA−AM (0.5 bar). (**d**) ZT−CFRP/MCFA−AM (0.5 bar). (**e**) No−pressure 3DP/CFRP samples.

The specimen dimensions were maintained to be the same for all laminate types, with a specimen thickness of 1.00 mm, a specimen width of 15.00 mm, and a specimen length of 80.00 mm. The measuring error was ±3% for the individual specimens. Figure 10 displays the compressive stress against the compressive strain (after the ASTM D695 recommended toe compensation procedure) plots for all five types of laminates. Note that the toe compensation procedure horizontally shifts the zero-strain point in order to project the linear portion extension of a curve (usually near the peak of the curve) to the origin (i.e., zero strain corresponds to zero stress) to filter out the artifacts causing the non-linear toe region at the beginning of a curve that is associated with seating or flattening the tilted ends of the specimen when the compression process starts; it can also filter out some other miscellaneous issues such as the minor specimen end partially crushed by the metal compression plates, but the specimen is not completely fractured by compression yet (it would look like a horizontal jump in the curve followed by a continuous rise of the curve). The curve patterns of ZT-CFRP samples (Figure 10b,d) clearly show more consistent longitudinal compressive strength values (among each type’s five specimens) compared with the curve patterns of the CFRP samples (Figure 10a,c,e). In fact, in terms of the longitudinal compressive strength (peak value of stress–strain curve), the ZT-CFRP specimens appear to be stronger and more consistent than the CFRP specimens when both are manufactured via the same process. This indicates that the CNF z-threads can improve the effectiveness of using carbon fiber’s compressive strength by preventing or delaying other premature failure modes that a traditional CFRP typically encounters (e.g., microfiber buckling, catastrophic delamination, crack propagation, etc.). On the other hand, according to the plots in Figure 10, the MCFA-AM specimens seem to have very similar longitudinal compressive strengths as the OOA-VBO specimens. This indicates the effectiveness of the magnetic compaction pressure for improving the longitudinal compressive strength. Further detailed comparisons in numbers will be discussed in a later section (also see the values in Appendix A).

### 3.2. Specimens’ Failure Analysis and Microscopic Morphology

**Figure 11:**
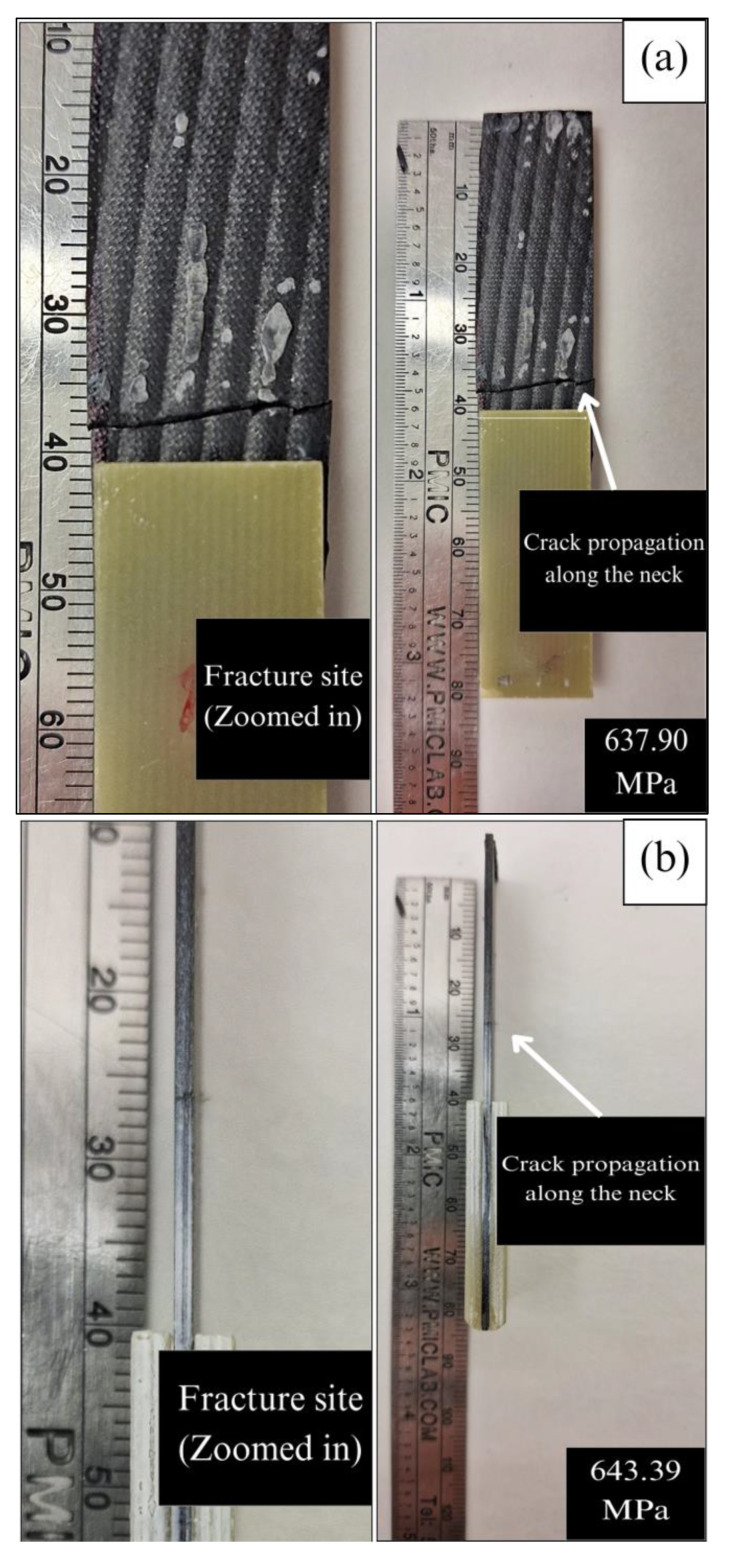

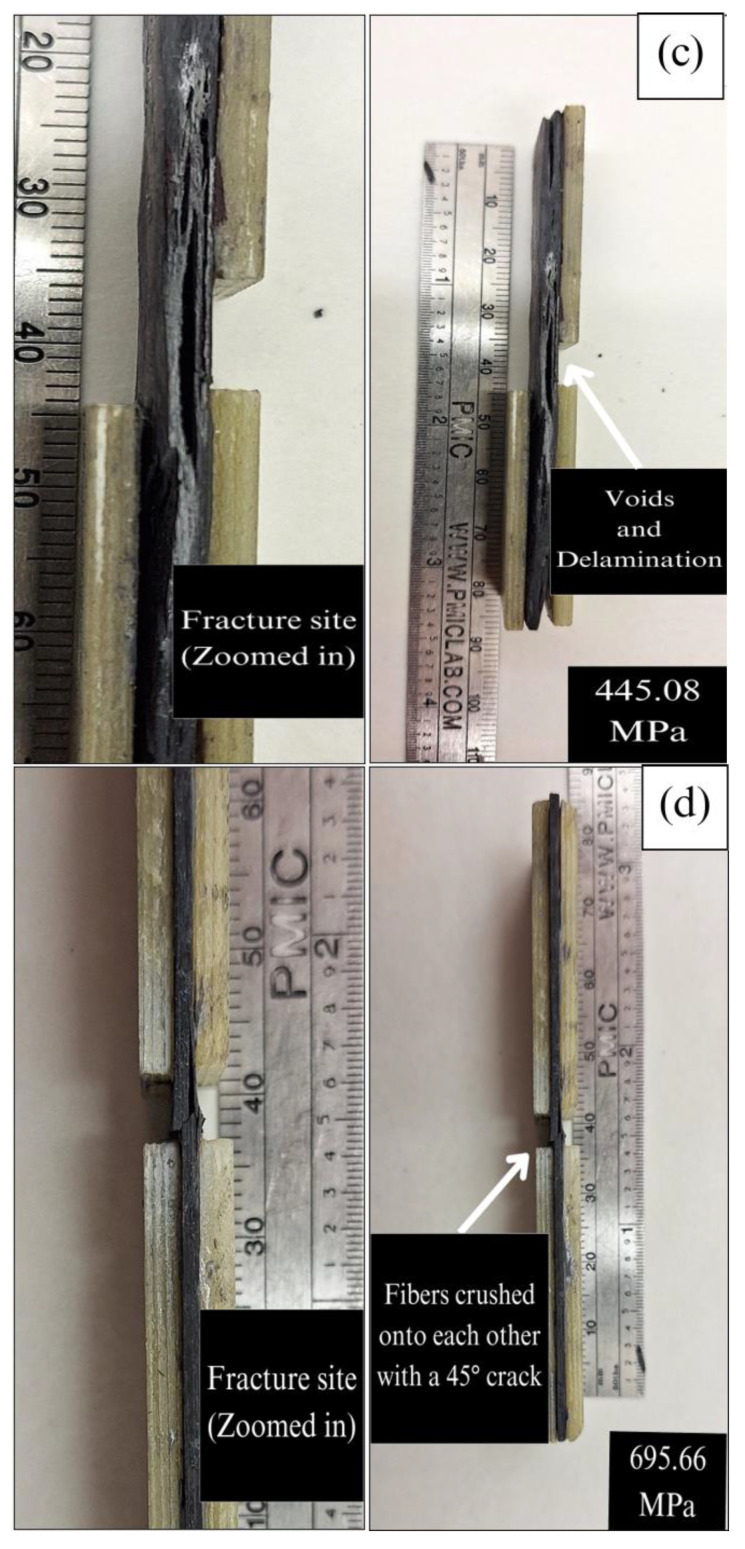

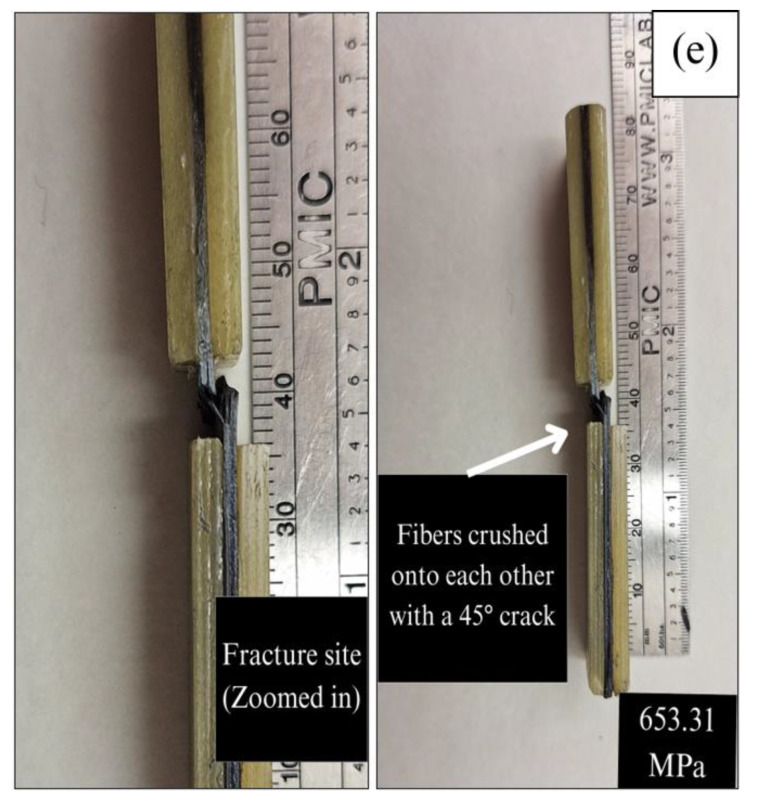
Fracture/failure sites of samples (with the fracture spot zoomed in), (**a**) CFRP/OOA−VBO, (**b**) CFRP/MCFA−AM (0.5 bar), (**c**) no−pressure 3DP/CFRP, (**d**) ZT−CFRP/OOA−VBO, (**e**) ZT−CFRP/MCFA−AM (0.5 bar).

The way in which a sample primarily undergoes failure due to compressive load is through fiber buckling, which is an acceptable mode of failure. Of the unacceptable modes of failure during testing before the testing’s final failure, tab debonding is one of the notable ones. To avoid this kind of failure, the tabs were bonded with J-B Weld^TM^, a toughened epoxy adhesive, to reinforce the bond-line with the testing specimen. Common types of failures after testing all samples were compressive failures, fiber breakage, and crushed ends. The failure sites and the zoomed in fracture spots of all different sample types are shown in Figure 11.

**Figure 12:**
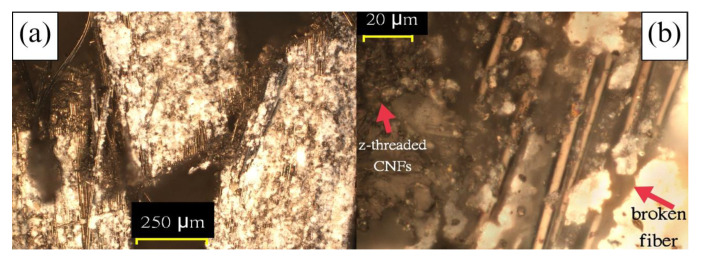
Microscope pictures of a ZT−CFRP/MCFA−AM sample, (**a**) (100×) top fiber crushing its way into the crack between bottom fibers due to compression, (**b**) (1000×) visible CNFs stitched in the z−direction.

**Figure 13:**
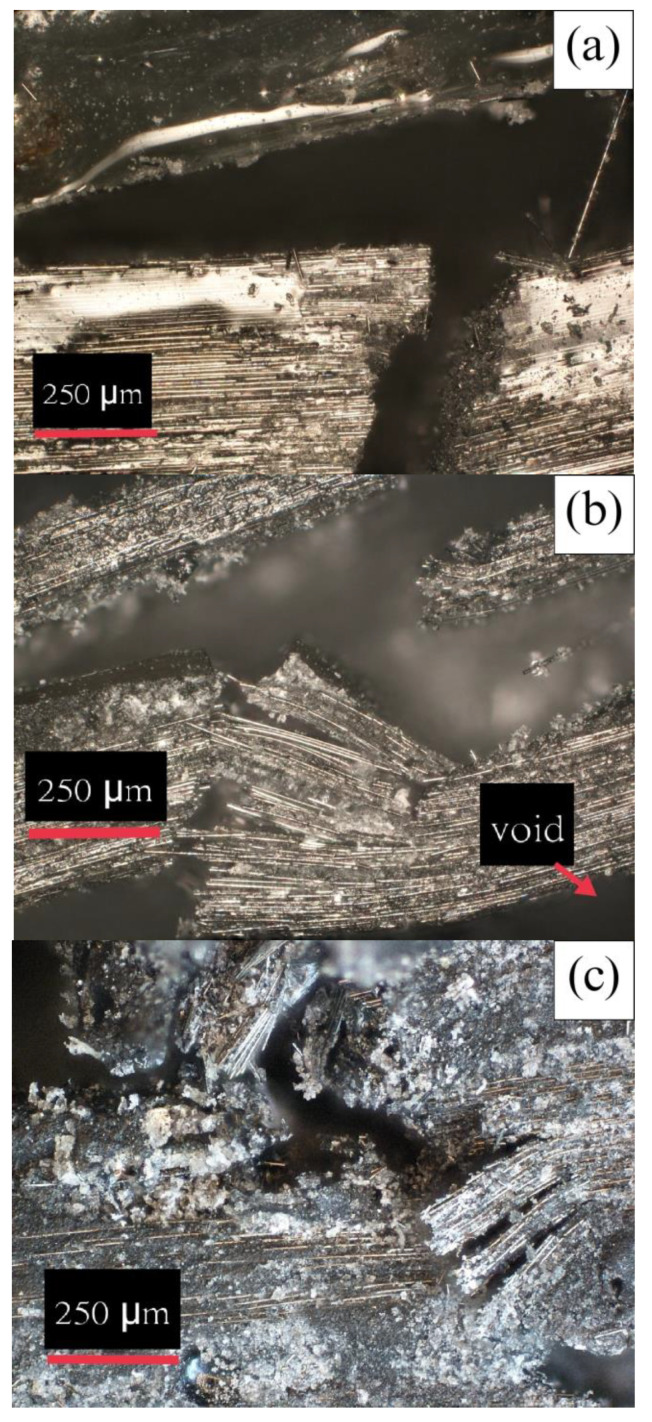
100× magnified microscope pictures, (**a**) CFRP/OOA−VBO sample, (**b**) ZT−CFRP/OOA−VBO sample, (**c**) CFRP/MCFA−AM sample.

Both the ZT-CFRP samples are observed to undergo a 45° crack, and the cracked portions are crushed onto each other. The CFRP samples underwent a natural fiber crack in the neck region with a crack propagation along the neck. Unlike the CFRP samples, the CNF z-threads impose resistance against delamination of the fibers and prevent crack propagation that keeps the sample from fiber failure before compressive failure. Lastly, the no-pressure 3DP sample went through both delamination and fiber failure due to the presence of voids and air traps. Microscope pictures of the failure and crack regions for all different samples are shown in Figure 12 and Figure 13. Pulled-off z-threaded CNFs from a ZT-CFRP sample are also visible in Figure 12b.

### 3.3. Discussion

The compressive strength of ZT-CFRP and regular CFRP samples cured by both MCFA-AM and OOA-VBO methods are put into comparison with respect to the benchmark control CFRP/OOA-VBO value (639.83 MPa) with a view to obtaining comprehensive knowledge of all the different laminate types produced in this study. Table 3 displays the mean longitudinal compressive strength values and other necessary data (mean) for all the samples used to run comparisons among one another (detailed testing data are provided in Appendix A).

**Figure 14:**
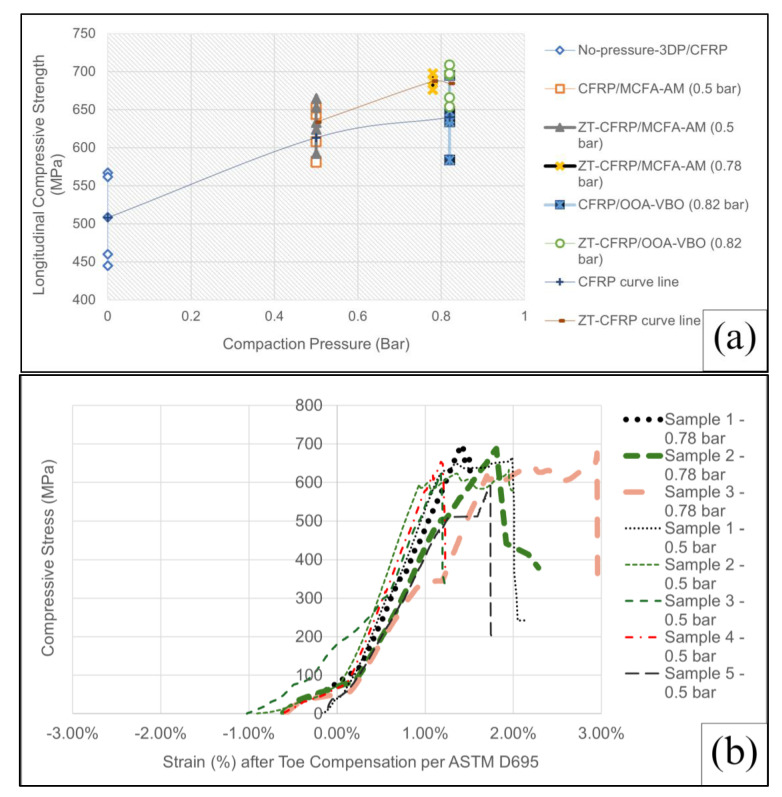
(**a**) Compressive strength of ZT−CFRP/MCFA-AM printed at 0.78 bar pressure in comparison to that of other samples. (**b**) Comparison of the stress–strain curves of ZT−CFRP/MCFA−AM samples printed at 0.5 bar, and 0.78 bar shows the improvement of compressive strength by increased compaction pressure of MCFA−AM for ZT−CFRP samples.

It is seen from the table that the coefficient of variation (COV) values for the ZT-CFRP samples are lower than that for the CFRP samples which establishes the reliability and better consistency of the ZT-CFRP samples. ZT-CFRP/MCFA-AM samples printed at 0.5 bar pressure (rolling compaction pressure) attained a mean strength of 633.43 MPa that is very close to that of the control CFRP/OOA-VBO (0.82 bar, static compaction pressure) samples. Therefore, the hypotheses are substantiated regarding how the reinforcement and interlocking mechanism of CNF z-threads enhanced the compressive strength. Additionally, new ZT-CFRP/MCFA-AM samples were produced (as per study [11]) at 0.78 bar pressure with a view to attaining improvement in the compressive strength with increased compaction pressure. The mean FVF was 55 ± 1%, the mean compressive strength was 687.30 MPa, and the COV was 2%. Figure 14a shows their compressive strength values (yellow dots) vs. compaction pressure with respect to the values of all other sample types. Figure 14b shows a comparison of the stress–strain curves of the ZT-CFRP/MCFA-AM samples printed at 0.5 bar and 0.78 bar; the results show the improvement of compressive strength by increasing the compaction pressure of MCFA-AM for the ZT-CFRP samples. It is evident from the comparison plot that compressive strength increased with the increase in the rolling compaction pressure from 0.5 bar to 0.78 bar.

In Figure 14a, there are two trendlines that are each specific to CFRP and ZT-CFRP test results, respectively. Both have similar behavior; the lines keep rising to a certain point and then become flat after intersecting the OOA-VBO samples printed at 0.82 bar. In Figure 14b, the slopes are within the same range, which means the higher rolling compaction pressure (0.78 bar) helped the ZT-CFRP further improve the ability of the carbon fibers against fiber buckling. While the micromechanics cannot be fully investigated by this experimental study, the experimental discovery shows that it would be of interest for future numerical analysis (such as finite element analysis) to further understand the micromechanics details, especially for CNF Z-threaded CFRP. According to [20], the SiO_2_ nanoparticles can help increase the elastic modulus of the epoxy matrix, thus helping stabilize the carbon fibers to delay the fiber micro-bucking under the longitudinal compression. Following the same rationale, one can also expect the CNF z-threads embedded inside a CFRP can enhance the elastic support given to the carbon fibers and mitigate fiber micro-buckling, and the elastic support provided by the CNF z-threads could likely be increased with a higher compaction pressure applied during the manufacturing process or a ZT-CFRP laminate with a lower resin content.

In the case of the MCFA-AM samples, we attempted to use 0.78 bar or even higher magnetic compaction pressure to print samples. However, it became difficult to detach the backing article by hand due to stronger magnetic compaction force in the current prototype printhead. As a result, for the current protype printing head, 0.78 bar is considered the optimum compaction pressure as of yet. The 0.78 bar ZT-CFRP/MCFA-AM samples (Table A5 shows detailed testing data) enhanced the compressive strength by 7.42% and 8.50% compared to CFRP/OOA-VBO (0.82 bar) and ZT-CFRP/MCFA-AM (0.5 bar), respectively. Furthermore, notably, the ZT-CFRP/MCFA-AM samples at 0.78 bar exhibited average compressive strength values that were very closely aligned with the ZT-CFRP/OOA-VBO (0.82 bar) samples (687.30 MPa compared to 698.19 MPa).

In practice, MCFA-AM was more capable of reducing the void content within the finished composite as every layer of prepreg was compressed one at a time under the same pressure while quickly curing. As for OOA-VBO, the void content within the composite part was manipulated by compressing all the piles of prepreg all at once over the course of its curing period. MCFA-AM currently is capable of producing simple geometries paired with a complex control system, whereas OOA-VBO is relatively simple in its operation. In terms of materials used during manufacturing, MCFA-AM surpasses OOA-VBO with fewer consumable materials (which are usually costly since they need to be able to withstand high temperature and the wear and tear during operation for several hours), smaller operating surfaces for post cleaning, and a much faster (about 2.7 times) production speed [11]. Since the MCFA-AM uses fewer consumable materials, it also better supports the sustainability of CFRP manufacturing than OOA-VBO. The nature of the MCFA-AM invention, according to the patent [13], due to the layer-by-layers deposition and the dynamic compaction pressure-controlled true 3D printing, will also allow in situ layer-by-layer quality inspection, repair, and each layer’s materials and compaction ratio adjustment, along with embedding sensors inside the composite parts for various functions (such as counterfeit protection, quality and health monitoring, assistant life-time maintenance and repair, etc.) during the MCFA-AM process. Different to other traditional composite manufacturing processes, the MCFA-AM was invented as a new generation manufacturing process with the Industry 4.0 and digital manufacturing in mind, trying to incorporate all the major processing parameters (such as pressure, temperature, curing, and void) deemed important to the quality and performance learned from traditional CFRP manufacturing processes. With further improvements to the control system and resin shelf life, MCFA-AM could meet or even surpass OOA-VBO in producing high-quality CFRP composites. Table 4 showcases the comparative compressive strength values of the unidirectional (0°, with an estimated uncertainty of ±5°) laminates from this paper and off-axial strength values of CF/nano-SiO_2_/epoxy composites [20]. Table 4 compares the longitudinal compressive strength values of unidirectional (0° ± 5°) laminates from this study with those of 2° and 10° off-axis misaligned carbon fiber/nano-SiO_2_/epoxy composites produced via vacuum-assisted resin transfer molding (VARTM) processes. These composites include varying volume fractions (0 vol%, 1.1 vol%, and 8.7 vol%) of nano-SiO_2_ to reinforce the matrix [20]. The comparison indicates that the longitudinal compressive strength of the 2° off-axis 8.7 vol% nano-SiO_2_-reinforced T700 CFRP/VARTM case is similar to, albeit slightly lower than, the AS4 CFRP/MCFA-AM (0.5 bar) case. It is noted that carbon fiber misalignments can significantly reduce the longitudinal compressive strength, as demonstrated by the 2° and 10° off-axis 8.7 vol% nano-SiO_2_-reinforced T700 CFRP/VARTM cases, where the strength dropped from 569.9 MPa to 352.1 MPa with a slight increase in the off-axis angle.

All the specimens in the study’s compressive tests could potentially have minor uncertainty in the off-axis misalignment (assumed to be less than 5°), which may not be easily detected by the naked eye. Additionally, Table 4 illustrates that increasing the nano-SiO_2_ concentration from 1.1 vol% to 8.7 vol% can enhance the 2° off-axis longitudinal compressive strength from 503.4 MPa to 569 MPa. This observation is similar to the findings in this study with the addition of CNF z-threads in a CFRP. However, the applied concentration (dosage) of CNF z-threads is significantly lower (1 wt%, which is approximately 0.5 vol% with respect to the matrix system).

## 4. Conclusions

Five distinct types of longitudinal samples were produced to assess compressive strength in comparison to the benchmark sample (CFRP/OOA-VBO at 0.82 bar pressure). Microscopic analysis was conducted on all variations in the samples to ascertain void contents and examine the morphology of fracture regions. This comprehensive analysis aimed to provide a better understanding of the testing and failure types exhibited by the different samples. ZT-CFRP/MCFA-AM samples were printed at two different compaction pressures (0.5 bar and 0.78 bar) to discover any improvement potential due to the increase in compaction pressure, and the average longitudinal compressive strength of ZT-CFRP/MCFA-AM (0.78 bar) samples exhibited a noticeable enhancement of 7.42% and 8.50% compared to the samples of CFRP/OOA-VBO (0.82 bar) and ZT-CFRP/MCFA-AM (0.5 bar), respectively.

The adjustment of compaction pressure and the reduction in voids not only impact longitudinal compressive strength but also influence various other mechanical and electrical properties in C-CFRP composites. A comprehensive study [11] on interlaminar shear strength, employing the same MCFA-AM method, resulted in sample laminates with a decreased void content. The ZT-CFRP/MCFA-AM (0.78 bar) samples exhibited an 11.34% improvement over control CFRP/OOA-VBO (0.82 bar) samples, attributable to the fiber interlocking capability of CNF z-threads. Furthermore, voids can have implications for the thermal properties of the composite, potentially diminishing thermal and electrical conductivity in the true thickness direction. Voids and air traps within the fiber laminate are less conductive compared to the carbon fiber and epoxy in the matrix. On the other hand, the CNF z-threads, even when voids are present in the composite, can still zigzag thread and interlock with the carbon fibers and mitigate the voids-induced adversary effects. This opens avenues for future research, focusing on exploring the effects of voids and the potential of ZT-CFRP technology in enhancing other properties of C-CFRP laminate parts.

The utilization of nanomaterials technology such as the CNF z-threading approach was observed to be effective in consistently enhancing the properties of composites. This improvement is attributed to the heightened resistance to microcrack growth at fracture spots and the facilitation of interlocking among the fiber arrays. Microscopic morphology images indicated the presence of pulled-off CNFs at the breakage zone, and from macroscopic fracture analysis, it was found that ZT-CFRP samples manufactured by both processes underwent compressive failure at the neck. However, CFRP and no-pressure samples underwent both types of failures (i.e., compressive failure and delamination). The novel MCFA-AM 3D printing process reduced the void content by a substantial extent by using magnetic compaction force and proved to be almost as effective as OOA-VBO’s void-control performance generated by more precise automatic vacuum suction. This innovative approach has not only increased the speed of laminate production by 2.7 [11] times compared to the OOA-VBO molding process but has also resulted in cost savings by reducing molding expenses, post-cleaning efforts, and mold-handling time. The ongoing development of the robotic MCFA-AM printer’s current prototype requires the integration of additional accessory components into its design. Significant progress in the operation and construction of the printer is expected by enhancing the coordination between printing speed, multi-stage curing control, dynamic magnetic compaction control, automated filament feeding, and automated control of anchor units. The expected future enhancement will make it feasible to manufacture laminates of higher quality in terms of performance, precision, and reliability.

## 5. Patents

This paper does not result in any patents. Nevertheless, it relies on the knowledge or methodologies outlined in the patents invented by the co-author/corresponding author:

- Hsiao, K.-T. Method and apparatus for 3D printing, US Patent No. US11426935B2, 30 August 2022. https://patents.google.com/patent/US11426935B2/en (also CN109843557B, EP3515690B1, JP6872268B2) (accessed on 3 April 2023);
- Hsiao, K.-T. Apparatus and method for directional alignment of nanofibers in a porous medium. US Patent No. US10556390B2, 11 February 2022 (https://patents.google.com/patent/US10556390B2/en) (also CN106660068B, EP3148711A4, JP6462115B2) (accessed on 3 April 2023).

## Figures and Tables

**Table 1:** Comparison of 3D-printed C-CFRP samples to those manufactured using either traditional manufacturing process or FDM 3D printing from the literature [9,10,11].

	3D-Printed Thermoplastic C-CFRP	3D-Printed Thermoset C-CFRP Examples		
Material	CF/Nylon [9]	CF/Epoxy (E-20) [10]	CF/Epoxy [11]	
			CFRP	ZT-CFRP
Fiber volume fraction (%)	35	48	53 ± 1	53 ± 1
Void (%)	3.0	2.5	1.36%	3.87%
Tensile strength (MPa)	1031	1476	N/A	N/A
Tensile strength change (%) vs. baseline value	+29%	−32%	N/A	N/A
Flexural strength (MPa)	945	858	N/A	N/A
Flexural strength change (%) vs. baseline value	+75%	−50%	N/A	N/A
ILSS (MPa)	N/A	45	62.83	69.73
ILSS change (%) vs. baseline value	N/A	−54%	+0.37% ^1^ +16.37% ^2^	+11.34% ^1^ +29.15% ^2^
Compressive strength (MPa)	N/A	N/A	N/A	N/A
3D printing methods found from the literature	3D compaction printing (3DCP); hot-compaction roller during FDM	Protrusion modified FDM pre-forms the sample, followed by powder resin melt infusion into the sample’s pores and post curing	MCFA-AM; a robotic MCFA-AM printing head; magnetic compaction pressure 0.78 bar	
Baseline printing methods compared in the literature	Filament manufacturer’s data sheet of samples printed with a typical FDM process	Carbon fiber manufacturer’s data sheet of samples produced by traditional autoclave process with full vacuum inside the vacuum bag (~−1 bar) and autoclave pressure 5.52–6.89 bars (85–100 psi) outside the vacuum bag	1. Traditional OOA-VBO process; full vacuum (~0.82 bar) applied inside the vacuum bag 2. No pressure FDM process	

**Table 2:** Cycle for curing in OOA−VBO method.

Time	Temperature (°C)	Vacuum Pump
20 min	30 °C	On
1 h	140 °C	On

**Table 3:** Mean longitudinal compressive strength results for all different laminate types.

Sample Type	Compaction Pressure (Bar)	Area (mm^2^) (Mean)	Ultimate Force (N) (Mean)	Ultimate Strain (%)	FVF (%)	Longitudinal Compressive Strength (MPa) (Mean)	COV (%)	Relative Improvement of Strength w.r.t. Control CFRP (%)
1 wt% ZT-CFRP /OOA-VBO	0.82 bar	16.13	11,251.16	1.19	54 ± 1	698.19	4%	+9.12%
Control CFRP/OOA-VBO	0.82 bar	15.27	9770.29	1.16	55 ± 1	639.83	6%	N/A
CFRP/MCFA-AM	0.5 bar	15.50	9488.34	1.12	54 ± 1	613.14	5%	−4.17%
No-Pressure 3DP/CFRP	0 bar	16.40	8325.17	1.16	35 ± 3	508.40	11%	−20.60%
1 wt% ZT-CFRP/MCFA-AM	0.5 bar	16.12	10,202.92	1.04	54 ± 1	633.43	4%	−1.00%
1 wt% ZT-CFRP/MCFA-AM	0.78 bar	16.11	11,076.28	1.09	55 ± 1	687.30	2%	+7.42%

Comment: the FVF was calculated from a sample picked randomly for all different types. The no-pressure 3DP sample (FVF 35 ± 3) picked randomly was the one with the lowest compressive strength.

**Table 4:** Compressive strength data for unidirectional and off−axial laminates.

Nanocomposite			Longitudinal Compressive Strength (MPa)		
**CNF** **(note: density of PR-24 CNF is about 2.1 g/cm^3^)**	**Percentage Amount**	**Case**	**0°** ± **5° (a**ssumed **u**ncertainty in off-axial carbon fiber misalignment)	**2°** off-axial carbon fiber misalignment	**10°** off-axial carbon fiber misalignment
	**1 wt%**	AS4 CFRP/OOA-VBO	639.83		
		AS4 ZT-CFRP/OOA-VBO	698.19		
		AS4 CFRP/No-pressure 3DP	508.40		
		AS4 CFRP/MCFA-AM (0.5 Bar)	613.14		
		AS4 ZT-CFRP/MCFA-AM (0.5 Bar)	633.43		
		AS4 ZT-CFRP/MCFA-AM (0.78 Bar)	687.30		
**Nano-SiO_2_** **(note: density of SiO_2_ is about 2.196 g/cm^3^)**	**0 vol%**	T700 CFRP/VARTM [20]	N/A	N/A	319.0 ± 20.3
	**1.1 vol%**		N/A	503.4 ± 47.5	324.1± 23.6
	**8.7 vol%**		N/A	569.9 ± 22.5	352.1 ± 19.2

## Data Availability

Detailed testing data are provided in Appendix A.

## Appendix A

**Table A1:** Compressive strength data for CFRP/OOA−VBO laminates.

Sample Order	Compaction Pressure	Thickness	Width	Area (mm^2^)	Ultimate Force (N)	FVF (%)	Ultimate Strength (MPa)	Failure Mode	Relative Compressive Strength Improvement w.r.t. Control CFRP (%)
Sample 1	0.82 bar	1.05	15.02	15.77	10,213.52	55 ± 1	647.61	Compressive failure	---
Sample 2	0.82 bar	1.00	14.85	14.85	9472.83	55 ± 1	637.90	Compressive failure	
Sample 3	0.82 bar	1.02	15.03	15.33	9737.37	55 ± 1	635.16	Compressive failure	
Sample 4	0.82 bar	1.01	15.01	15.16	10531.7	55 ± 1	694.70	Compressive failure	
Sample 5	0.82 bar	1.02	14.94	15.24	8896.02	55 ± 1	583.77	Compressive failure	
Mean		1.02	14.98	15.27	9770.29	55 ± 1	639.83		
STDEV		0.02	0.08	0.33	638.38		39.49		
Minimum		1.00	14.85	14.85	8896.02		583.77		
Maximum		1.05	15.03	15.77	10,531.70		694.70		
COV (%)		2%	1%	2%	7%		6%		

**Table A2:** Compressive strength data for ZT−CFRP/OOA-VBO laminates.

Sample Order	Compaction Pressure	Width	Thickness	Area (mm^2^)	Ultimate Force (N)	FVF (%)	Ultimate Strength (MPa)	Failure Mode	Relative Compressive Strength Improvement w.r.t. Control CFRP (%)
Sample 1	0.82 bar	15.05	1.07	16.10	11,413.31	54 ± 1	708.75	Compressive failure	+9.12%
Sample 2	0.82 bar	15.05	1.12	16.86	11,725.97	54 ± 1	695.66	Compressive failure	
Sample 3	0.82 bar	15.03	1.10	16.53	10,811.82	54 ± 1	653.95	Compressive failure	
Sample 4	0.82 bar	15.08	1.04	15.68	11,011.42	54 ± 1	702.11	Compressive failure	
Sample 5	0.82 bar	15.01	1.03	15.46	11,293.30	54 ± 1	730.47	Compressive failure	
Mean		15.04	1.09	16.13	11,251.16	54 ± 1	698.19		
STDEV		0.02	0.03	0.58	354.97		27.98		
Minimum		15.01	1.05	15.46	10,811.82		653.95		
Maximum		15.05	1.13	16.86	11,725.97		730.47		
COV (%)		0%	3%	4%	3%		4%		

**Table A3:** Compressive strength data for CFRP/MCFA−AM (0.5 bar) laminates.

Sample Order	Compaction Pressure	Width	Thickness	Area (mm^2^)	Ultimate Force (N)	FVF (%)	Ultimate Strength (MPa)	Failure Mode	Relative Compressive Strength Improvement w.r.t. Control CFRP (%)
Sample 1	0.5 bar	15.03	1.01	15.18	9766.90	54 ± 1	643.39	Compressive Failure	−4.17
Sample 2	0.5 bar	15.01	1.04	15.61	9088.00	54 ± 1	582.18	Compressive Failure	
Sample 3	0.5 bar	15.0	1.03	15.45	8969.41	54 ± 1	580.54	Fiber Breakage	
Sample 4	0.5 bar	15.03	1.01	15.18	9224.96	54 ± 1	607.69	Fiber Breakage	
Sample 5	0.5 bar	15.04	1.06	15.94	10392.43	54 ± 1	651.87	Compressive Failure	
Mean		15.02	1.03	15.5	9488.34	54 ± 1	613.14		
STDEV		0.02	0.02	0.32	590.32		33.42		
Minimum		15.00	1.01	15.2	8969.41		580.54		
Maximum		15.04	1.06	15.9	10392.43		651.87		
COV (%)		0%	2%	2%	6%		5%		

**Table A4:** Compressive strength data for ZT−CFRP/MCFA−AM (0.5 bar) laminates.

Sample Order	Compaction Pressure	Width	Thickness	Area (mm^2^)	Ultimate Force (N)	FVF (%)	Ultimate Strength (MPa)	Failure Mode	Relative Compressive Strength Improvement w.r.t. Control CFRP (%)
Sample 1	0.5 bar	15.07	1.02	15.37	10,217.61	54 ± 1	664.72	Compressive Failure	−1.00%
Sample 2	0.5 bar	15.05	1.11	16.70	10,572.02	54 ± 1	632.85	Compressive Failure	
Sample 3	0.5 bar	15.05	1.08	16.25	10,142.63	54 ± 1	624.01	End Crushed	
Sample 4	0.5 bar	15.02	1.05	15.77	10,303.30	54 ± 1	653.31	Compressive Failure	
Sample 5	0.5 bar	15.01	1.1	16.51	9779.04	54 ± 1	592.27	Compressive Failure	
Mean		15.04	1.07	16.12	10,202.92	54 ± 1	633.43		
STDEV		0.02	0.04	0.546	287.16		28.09		
Minimum		15.01	1.02	15.37	9779.04		592.27		
Maximum		15.07	1.11	16.70	10,572.02		664.72		
COV (%)		0%	3%	3%	3%		4%		

**Table A5:** Compressive strength data for ZT−CFRP/MCFA−AM (0.78 bar) laminates.

Sample Order	Compaction Pressure	Width	Thickness	Area (mm^2^)	Ultimate Force (N)	FVF (%)	Ultimate Strength (MPa)	Failure Mode	Relative Compressive Strength Improvement w.r.t. Control CFRP (%)
Sample 1	0.78 bar	15.1	1.05	15.86	11,058.08	55% ± 1	697.22	Compressive Failure	+7.42%
Sample 2	0.78 bar	15.17	1.08	16.38	11,276.94	55% ± 1	688.46	Compressive Failure	
Sample 3	0.78 bar	15.2	1.06	16.11	10,893.83	55% ± 1	676.22	Compressive Failure	
Mean		15.16	1.06	16.11	11,076.28	55% ± 1	687.30		
STDEV		0.05	0.02	0.26	192.20		10.55		
Minimum		15.10	1.05	15.86	10,893.83		676.22		
Maximum		15.20	1.08	16.38	11,276.94		697.22		
COV (%)		0%	1%	1%	2%		2%		

Comment: These three data points were additional tests to check that raising the compaction pressure can increase the compressive strength as linearly approximated and all three data points were in close proximity to each other. Thus, there was no need for five data points.

**Table A6:** Compressive strength data for no−pressure 3DP CFRP laminates.

Sample Order	Compaction Pressure	Width	Thickness	Area (mm^2^)	Ultimate Force (N)	FVF (%)	Ultimate Strength (MPa)	Failure Mode	Relative Compressive Strength Improvement w.r.t. Control CFRP (%)
Sample 1	0 bar	15.05	1.07	16.10	9127.85	35 ± 3	566.82	Compressive failure	−20.60%
Sample 2	0 bar	15.05	1.12	16.85	7502.23	35 ± 3	445.08	Compressive failure	
Sample 3	0 bar	15.03	1.10	16.53	7605.21	35 ± 3	460.00	Compressive failure	
Sample 4	0 bar	15.04	1.07	16.09	9036.52	35 ± 3	561.53	Fiber breakage	
Sample 5	0 bar	15.07	1.09	16.43	8354.05	35 ± 3	508.58	Fiber breakage	
Mean		15.05	1.09	16.40	8325.17	35 ± 3	508.40		
STDEV		0.01	0.02	0.319	765.95		56.10		
Minimum		15.03	1.07	16.09	7502.23		445.08		
Maximum		15.07	1.12	16.85	9127.85		566.82		
COV (%)		0%	2%	1%	9%		11%		

**Table A7:** Statistical analysis of the microscopy images of voids taken at 100× magnification.

No. of Image Spots	CFRP (MCFA-AM) Void Area πab (µm^2^)	ZT-CFRP (MCFA-AM) Void Area πab (µm^2^)	No Pressure CFRP Void Area πab (µm^2^)	Control CFRP (OOA-VBO) Void Area πab (µm^2^)	ZT-CFRP (OOA-VBO) Void Area πab (µm^2^)
1	42,093.33	12,937.984	250,000	79,023.38	13,022.84
2	12,938.57	76,320.390	247,123.89	19,422.06	46,379.10
3	0	0	170,685.83	0	10,167.73
Maximum	42,093.33	76,320.390	250,000	79,023.38	46,379.10
Minimum	0	0	170,685.83	0	10,167.73
Average void area	18,343.67	29,752.34	222,602.67	32,815	23,189.89
Total area	10.0 × 10^5^ µm^2^	10.0 × 10^5^ µm^2^	10.0 × 10^5^ µm^2^	10.0 × 10^5^ µm^2^	10.0 × 10^5^ µm^2^
Average void content	1.83%	2.98%	22.26%	3.28%	2.32%
